# Variation in Influenza B Virus Epidemiology by Lineage, China

**DOI:** 10.3201/eid2408.180063

**Published:** 2018-08

**Authors:** Juan Yang, Yiu Chung Lau, Peng Wu, Luzhao Feng, Xiling Wang, Tao Chen, Sheikh T. Ali, Zhibin Peng, Vicky J. Fang, Juanjuan Zhang, Yangni He, Eric H.Y. Lau, Ying Qin, Jing Yang, Jiandong Zheng, Hui Jiang, Hongjie Yu, Benjamin J. Cowling

**Affiliations:** Chinese Center for Disease Control and Prevention, Beijing, China (Juan Yang, L. Feng, T. Chen, Z. Peng, Y. Qin, Jing Yang, J. Zheng, H. Jiang, H. Yu);; Fudan University School of Public Health, Key Laboratory of Public Health Safety, Ministry of Education, Shanghai, China (Juan Yang, X. Wang, J. Zhang, Y. He, H. Yu);; The University of Hong Kong, Hong Kong, China (Y.C. Lau, P. Wu, S.T. Ali, V.J. Fang, E.H.Y. Lau, B.J. Cowling)

**Keywords:** Influenza B, epidemiology, lineage, Yamagata, Victoria, China, viruses, influenza, respiratory infections

## Abstract

We used national sentinel surveillance data in China for 2005–2016 to examine the lineage-specific epidemiology of influenza B. Influenza B viruses circulated every year with relatively lower activity than influenza A. B/Yamagata was more frequently detected in adults than in children.

Influenza B virus, first identified in 1940 (*1*), is associated with considerable hospital admissions and deaths worldwide every year (*2*). During the early 1980s, influenza B viruses split into 2 lineages, termed B/Victoria and B/Yamagata (*3*). These 2 lineages showed distinct antigenicity and transmission dynamics (*4*) and have co-circulated during each influenza season since 2001 (*2*). Relatively less attention has been given to influenza B virus epidemiology than to influenza A epidemiology (*2*) because influenza B virus spreads almost exclusively in humans and does not pose a pandemic threat (*5*).

Several recent reports have highlighted potential differences in the epidemiology of B/Victoria and B/Yamagata lineage viruses, including younger average ages of persons with B/Victoria virus infection (*4*,*6*,*7*) and greater transmissibility of B/Victoria viruses (*4*,*6*). Our study aimed to describe epidemiologic patterns of influenza B virus activity in China and to identify and compare the seasonality and age distribution of persons with medically attended influenza B/Victoria and B/Yamagata virus infections.

## The Study

The Chinese Center for Disease Control and Prevention coordinated influenza surveillance in sentinel clinics during October 2005–March 2016. Sentinel hospitals in provinces in southern China conducted year-round surveillance; in northern China (except for Liaoning, Gansu, and Tianjin provinces, where year-round surveillance was conducted), surveillance was suspended from April to September before 2009 because influenza has low activity in summer in these temperate areas of China (*8*). Sentinel surveillance was then expanded from 193 to 554 hospitals conducting year-round surveillance in all provinces since 2009. Sentinel hospitals reported the number of outpatients and the number of outpatients with influenza-like illness symptoms on a daily basis. Respiratory specimens collected from a subset of outpatients with influenza-like illness were tested for influenza viruses. Each sentinel hospital in northern China was required to collect 10–15 samples per week during October–March and 5–15 samples per month during April–September for virus testing, and the hospitals in southern China tested 5–15 samples per week throughout the year. Most laboratories had adopted real-time PCR for lineage identification since 2009; some laboratories still use virus culture followed by hemagglutination inhibition test (Technical Appendix). Individual data on age, sex, and date of specimen collection also were reported for all selected patients for virus testing (Technical Appendix). Because national influenza sentinel surveillance was part of a routine public health investigation, the study was exempt from institutional review board assessment, and all data were delinked from identifiable personal information.

We used a proxy measure of influenza activity in the communities served by the sentinel locations because it was previously indicated to be a good correlate of the incidence rates of influenza virus infection in the community (*9*). The proxy was calculated as the product of the weekly rates for influenza-like illness consultation and the proportion of sentinel specimens testing positive for each lineage in the same week. The age-specific proportions of sentinel specimens testing positive for influenza B virus by lineage were derived as the proportion of sentinel specimens testing positive for each lineage (numerator) among the outpatients recruited for specimen collection (denominator) by exact year of age.

**Figure 1:**
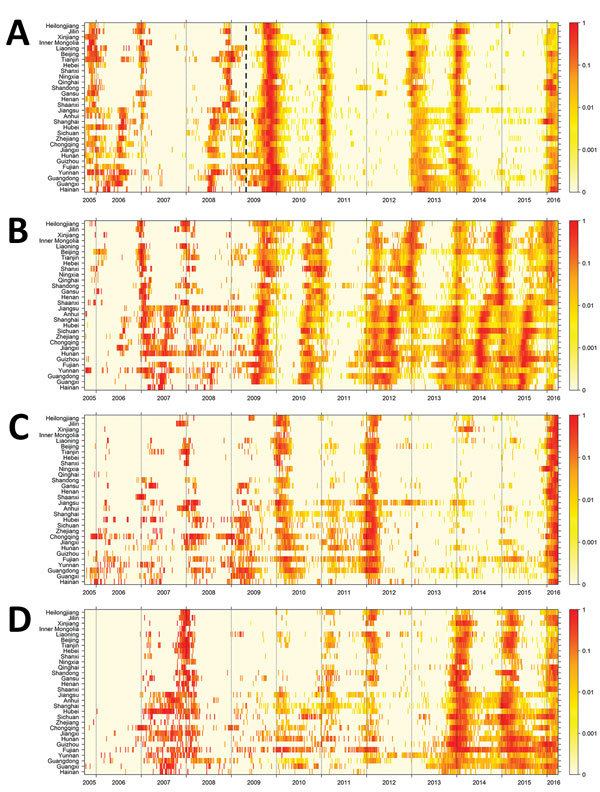
Heatmap of influenza virus activity by lineage in 30 provinces and municipalities (sorted by latitude), China, October 2005–March 2016. A) Influenza A(H1N1); B) influenza A(H3N2); C) influenza B Victoria lineage; D) influenza B Yamagata lineage. Map is based on 2,498,735 specimens collected from the sentinel hospitals. Normalized virus activity is shown for each province and municipality as the product of the weekly proportion of influenza-like illness consultations and the weekly proportion of sentinel specimens testing positive for influenza viruses divided by the maximum virus activity in the province or municipality throughout the study period to give a rescaled proxy with values between 0 (no activity) and 1 (highest activity in that province). The dashed line in panel for A indicates the start of the H1N1 pandemic in 2009.

We found that influenza B/Victoria and B/Yamagata lineages circulated every year in mainland China during 2005–2016 and were mostly active during the winter–spring seasons (Figure 1). Influenza B virus activity was generally less intense than influenza A activity and less apparent during the 2005–06, 2010–11, and, particularly, 2012–13 seasons (Figure 1; Technical Appendix Figure 1).

Influenza B/Victoria activity increased in every season before and during the first wave of infections with influenza A(H1N1)pdm09 virus in China in late 2009, whereas substantial virus detections were only seen in the early 2011–12 and 2015–16 seasons during the postpandemic period. B/Yamagata lineage led to 3 major epidemics during the 2007–08, 2013–14, and 2014–15 seasons (Technical Appendix Figure 1). These major epidemics were associated with prolonged influenza activity, particularly during summer periods and in provinces and municipalities with lower latitude, which occurred during 2008–2011 for B/Victoria lineage and during the 2007–08 and 2014–15 seasons for B/Yamagata lineage (Figure 1).

**Figure 2:**
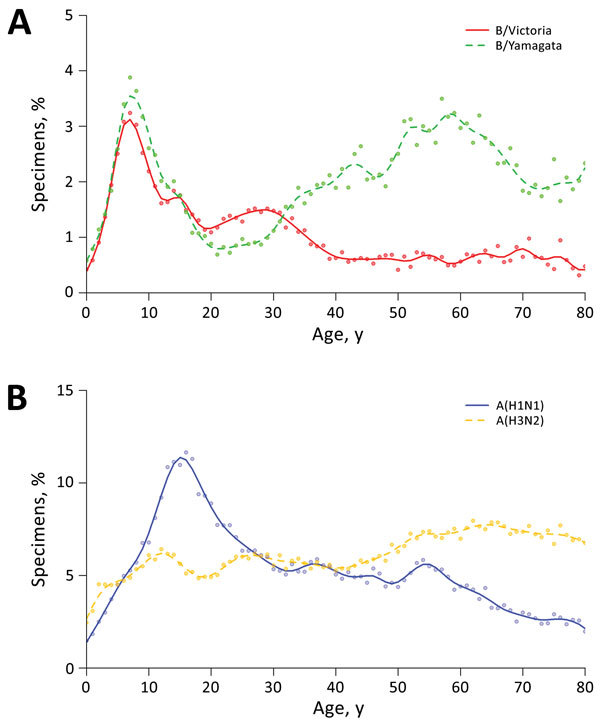
Age-specific proportions of sentinel specimens collected from sentinel surveillance sites testing positive for influenza, China, October 2005–March 2016. A) Influenza B/Victoria and B/Yamagata lineages; B) influenza A(H1N1) and A(H3N2). Findings are based on 2,498,735 specimens collected from the sentinel hospitals. Dots indicate the original data, and lines (solid and dashed) show the estimation from a fitted smoothing function to the pattern by age.

Children 5–15 years of age had the highest detection rates among all age groups for both lineages. The rates of detection of B/Victoria lineage viruses decreased with age after peaking at 10 years of age, and the rates of B/Yamagata lineage virus infections generally increased among persons >25 years of age to a second peak in older adults (Figure 2, panel A). The patterns differed somewhat across provinces and municipalities without systematic variation by latitude (Technical Appendix Figure 2). In comparison, influenza A(H1N1) showed an age pattern similar to that for B/Victoria but with a later peak, at 10–20 years of age; however, influenza A(H3N2) indicated largely comparable virus detections across different age groups (Figure 2, panel B).

## Conclusions

Our study showed that influenza B virus generally was relatively less active than influenza A virus (Figure 1; Technical Appendix Figure 1). Influenza B/Yamagata caused fewer epidemics than B/Victoria during the study period, largely consistent with findings from a study using sentinel surveillance data from multiple countries (*10*). The alternating predominance of the B/Victoria and B/Yamagata lineages, especially after 2009, and the low influenza B virus activity in China during the 2012–13 season might reflect the complex interactions between population immunity and virus evolution of influenza B lineages (*11*).

Our study suggested a potential difference in the age patterns of persons infected with B/Yamagata and B/Victoria (Figure 2). The elevated proportions of infections with both lineages in children might indicate a lack of exposure to the virus early in life (*4*,*12*). However, the discrepancy in susceptibility to infections with B/Victoria and B/Yamagata in older adults might reflect the genetic difference in viruses of the 2 lineages, although previous exposure to different lineages and vaccination history might have had an effect. Antigenic analysis indicated that circulating B/Yamagata strains in general showed a larger genetic diversity than B/Victoria strains (*4*). This genetic diversity may lead to a substantial number of persons infected with a certain strain of B/Yamagata virus who are susceptible to the other co-circulating strains of the same lineage. The declining frequency of B/Victoria detections with age, however, implied a gradually strengthened immunity in older persons, which could be attributed to accumulated immunity from exposure to virus strains with fewer genetic changes or possibly to the boosted heterologous immunity against B/Victoria viruses induced by exposure to B/Yamagata viruses (*13*).

The study has several limitations. First, expansion of the national sentinel surveillance system in China since 2009 might have affected the observed patterns in virus activity because of inclusion of sentinel clinics providing healthcare services specifically to certain populations, such as patients in respiratory or pediatric outpatient clinics, although we weighted virus activity by age in the analysis. Second, the wider application of PCR in national surveillance laboratories might have led to an artificial increase in virus activity; however, we assumed that this change would not differ between the 2 lineages.

Further work could examine the degree of cross-protection conferred by infections of the opposite lineage, if any (*13*,*14*). Results from such studies would further elucidate the epidemiology of influenza B virus and optimize vaccination strategies in China.

Technical Appendix National influenza sentinel surveillance, China; laboratory methods for determining influenza B lineages; proportion of clinics involved in national influenza sentinel surveillance; distribution of specimens from surveillance hospitals and proportions of network laboratories using various testing methods; national influenza virus activity by virus subtype; and patient age–specific proportions of specimens testing positive for influenza B/Victoria and B/Yamagata lineages.
