# Supplementary material for: Variation in Influenza B Virus Epidemiology by Lineage, China
Source: Emerg Infect Dis. 2018 Aug;24(8):1536–40. doi: 10.3201/eid2408.180063 (PMC6056115; doi:10.3201/eid2408.180063)
Supplement: Technical Appendix — National influenza sentinel surveillance, China; laboratory methods for determining influenza B lineages; proportion of clinics involved in national influenza sentinel surveillance; distribution of specimens from surveillance hospitals and proportions of network laboratories using various testing methods; national influenza virus activity by virus subtype; and patient age–specific proportions of specimens testing positive for influenza B/Victoria and B/Yamagata lineages. [file 18-0063-Techapp-s1.pdf]

Article DOI: https://doi.org/10.3201/eid2408.180063

Variation in Influenza B Virus Epidemiology by Lineage, China

Technical Appendix

National Influenza Sentinel Surveillance in China

The influenza sentinel surveillance in China was established in 2005 and has been substantially expanded from 193 to 554 sentinel hospitals since the 2009 influenza A(H1N1)pdm09 pandemic. The surveillance has involved multiple outpatient clinics in sentinel hospitals, including internal medicine, internal medicine emergency, pediatrics, pediatric emergency and fever clinics. The number of clinics would change over time because more sentinel hospitals have been included into the surveillance, and a small proportion of sentinel hospitals might have been removed from the surveillance system. The annual proportions of clinics involved in the surveillance are shown in Technical Appendix Table 1.

Laboratory Methods for Determining Influenza B Lineages

The national influenza sentinel surveillance in China currently comprises 560 sentinel hospitals and has been substantially expanded since the 2009 influenza A(H1N1) pandemic. The network laboratories in the surveillance system conducted virus determination according to the standard methods in the World Health Organization (WHO) protocols of the Manual for the Laboratory Diagnosis and Virological Surveillance of Influenza (*1*) and WHO Information for the Molecular Detection of Influenza Viruses (*2*).

Before the 2009 A(H1N1) pandemic, influenza B lineages were differentiated by virus culture followed by the hemagglutination-inhibition (HAI) assay with standardized antiserum. To monitor the circulating characteristics of 2 influenza B virus lineages rapidly and sensitively, real-time reverse transcription PCR (rRT-PCR) began to be used more frequently since May 2009, when the A(H1N1) pandemic occurred. Since then the laboratories in the surveillance

network have been performing either virus culture followed by HAI assay or rRT-PCR or both for lineage determination.

1. Virus Culture Followed by HAI Assay

Samples are inoculated into MDCK (ATCC CCL 34) cells and/or specific pathogen-free embryonated chicken eggs (9–11 days old) for virus isolation in biosafety level 2 facilities at the influenza surveillance network laboratories. MDCK cells were grown in Dulbecco's modified Eagle's medium (Invitrogen, Carlsbad, CA, USA) containing 10% fetal bovine serum (Invitrogen). In the HAI assays, the standardized antigen and ferret antiserum that raised against B/Yamagata and B/Victoria lineages recommended by WHO for Northern Hemisphere influenza vaccine strains were prepared by the Chinese National Influenza Center (CNIC), one of WHO's influenza reference center, following the WHO Manual for the Laboratory Diagnosis and Virological Surveillance of Influenza (*1*). The standardized antigen and ferret antiserum were provided to all influenza surveillance network laboratories by CNIC. The details of virus culture followed by HAI assay to determine influenza B lineage have been published by the authors elsewhere (*3*). The quality control and assessment for the virus isolation and lineages differentiation were operated by CNIC yearly.

2. 1-Step rRT-PCR for the Detection of Influenza B Lineage

Viral RNA was extracted using the QIAamp Viral RNA Minikit (QIAGEN, Hilden, Germany, cat.no. 52904) according to the manufacturer's instructions. One-step rRT-PCR was further used to detect influenza B type and determine lineages. The primers and probes sequence were provided by CNIC to all influenza surveillance network laboratories according to the WHO Information for the Molecular Detection of Influenza Viruses (*2*). The reaction master mixture and thermocycling conditions also was performed according to (*2*). A cycle threshold $\leq$37 was considered positive for the influenza B lineage determination. Several predominant influenza B virus strains, including B/Yamagata and B/Victoria in annual influenza epidemics, were used to validate the sensitivity of primers and probes. Several influenza A virus strains, including seasonal H1N1, pandemic H1N1, seasonal H3N2; and avian influenza H5N1, H9N2, and H7N9 (since 2013) were used to validate the specificity of the assays by CNIC yearly. The sequence of primers and probes with no cross-reactivity reactions would be provided to network laboratories

for the lineage detection. All the 1-step rRT-PCRs were performed by network laboratories. The quality control and assessment for the lineages differentiation were operated by CNIC yearly.

Technical Appendix Table 2 reports the distributions of specimens by age group, province, and testing method in each year of the study period.

References

1. World Health Organization Global Influenza Surveillance Network. Manual for the laboratory diagnosis and virological surveillance of influenza [cited 2018 Mar 21]. http://www.who.int/influenza/gisrs_laboratory/manual_diagnosis_surveillance_influenza/en/

2. World Health Organization. Information for the molecular detection of influenza viruses [cited 2018 Mar 21]. http://www.who.int/influenza/gisrs_laboratory/WHO_information_for_the_molecular_detection_of_influenza_viruses_20171023_Final.pdf

3. Du X, Dong L, Lan Y, Peng Y, Wu A, Zhang Y, et al. Mapping of H3N2 influenza antigenic evolution in China reveals a strategy for vaccine strain recommendation. Nat Commun. 2012;3:709. PubMed http://dx.doi.org/10.1038/ncomms1710

Technical Appendix Table 1. Annual proportions of clinics involved in the national influenza sentinel surveillance, China*

	Clinic					
Season	Internal medicine	Internal medicine emergency	Pediatrics	Pediatric emergency	Fever clinic	Total no. sentinel clinics
2005–06	0.249	0.213	0.301	0.165	0.071	162108
2006–07	0.246	0.212	0.296	0.163	0.084	184370
2007–08	0.243	0.209	0.297	0.165	0.085	187625
2008–09	0.232	0.205	0.276	0.154	0.132	262621
2009–10	0.220	0.206	0.245	0.140	0.188	619799
2010–11	0.225	0.209	0.248	0.139	0.180	669101
2011–12	0.225	0.210	0.247	0.143	0.176	699090
2012–13	0.223	0.210	0.247	0.145	0.175	715918
2013–14	0.223	0.209	0.246	0.145	0.177	723199
2014–15	0.222	0.210	0.246	0.147	0.175	727677
2015–16	0.221	0.210	0.246	0.149	0.175	372462

*In general, the proportions of sentinel clinics by type of clinic remained relatively stable during the study period, whereas more fever clinics have been involved in the surveillance more recently.

Technical Appendix Table 2. Distribution of sentinel specimens from sentinel surveillance hospitals and proportions of network laboratories using different testing methods in each influenza season, China

	Influenza season										
Variable	2005–06	2006–07	2007–08	2008–09	2009–10	2010–11	2011–12	2012–13	2013–14	2014–15	2015–16
Total no. specimens	37,360	39,024	41,540	141,825	321,859	201,652	219,633	301,080	442,314	462,756	289,692
Age group, y, %											
0–5	0.512	0.542	0.548	0.341	0.307	0.432	0.433	0.45	0.444	0.474	0.48
6–10	0.173	0.145	0.141	0.129	0.132	0.117	0.125	0.106	0.121	0.117	0.133
11–15	0.057	0.055	0.05	0.102	0.096	0.052	0.056	0.047	0.045	0.042	0.039
16–20	0.051	0.05	0.048	0.107	0.107	0.058	0.055	0.053	0.049	0.042	0.039
21–25	0.048	0.046	0.047	0.081	0.09	0.073	0.066	0.067	0.059	0.051	0.043
26–30	0.04	0.044	0.042	0.067	0.076	0.068	0.065	0.068	0.064	0.06	0.059
31–35	0.03	0.027	0.025	0.039	0.04	0.04	0.04	0.042	0.041	0.038	0.039
36–40	0.022	0.021	0.023	0.034	0.034	0.035	0.031	0.032	0.032	0.028	0.027
41–45	0.016	0.015	0.015	0.022	0.023	0.025	0.024	0.026	0.027	0.025	0.024
46–50	0.013	0.013	0.014	0.02	0.022	0.025	0.026	0.026	0.025	0.025	0.024
51–55	0.012	0.012	0.013	0.017	0.019	0.02	0.019	0.018	0.021	0.023	0.022
56–60	0.009	0.009	0.01	0.015	0.017	0.02	0.02	0.021	0.023	0.023	0.019
61–65	0.005	0.007	0.006	0.008	0.011	0.011	0.013	0.015	0.017	0.018	0.017
66–70	0.004	0.005	0.006	0.006	0.008	0.008	0.009	0.01	0.011	0.012	0.012
71–75	0.004	0.004	0.004	0.006	0.007	0.007	0.008	0.008	0.009	0.009	0.008
76–80	0.003	0.003	0.004	0.004	0.006	0.006	0.006	0.007	0.008	0.008	0.007
>80	0.002	0.002	0.002	0.003	0.004	0.004	0.004	0.005	0.006	0.007	0.007
Province, %											
Heilongjiang	0.01	0.016	0.022	0.028	0.025	0.033	0.039	0.031	0.032	0.03	0.039
Jilin	0.018	0.028	0.025	0.017	0.023	0.018	0.018	0.019	0.019	0.019	0.025
Xinjiang	0.019	0.014	0.014	0.022	0.037	0.04	0.032	0.028	0.029	0.028	0.039
Inner Mongolia	0.008	0.017	0.021	0.021	0.021	0.018	0.019	0.021	0.02	0.022	0.032
Liaoning	0.022	0.018	0.023	0.043	0.063	0.037	0.037	0.037	0.033	0.03	0.039
Beijing	0.026	0.016	0.017	0.032	0.033	0.035	0.049	0.037	0.027	0.025	0.02
Tianjin	0.027	0.026	0.026	0.013	0.012	0.015	0.015	0.014	0.014	0.013	0.017
Hebei	0.029	0.024	0.024	0.038	0.034	0.029	0.033	0.035	0.029	0.034	0.046
Shanxi	0.006	0.013	0.016	0.017	0.014	0.018	0.02	0.022	0.022	0.023	0.033
Ningxia	0.012	0.011	0.012	0.012	0.019	0.012	0.011	0.014	0.013	0.014	0.017
Qinghai	0.014	0.009	0.009	0.006	0.006	0.011	0.009	0.012	0.009	0.013	0.014
Shandong	0.017	0.02	0.023	0.025	0.037	0.047	0.045	0.046	0.044	0.039	0.051
Gansu	0.035	0.043	0.046	0.041	0.028	0.032	0.032	0.029	0.024	0.027	0.035
Henan	0.011	0.018	0.015	0.048	0.033	0.029	0.03	0.035	0.033	0.032	0.042
Shaanxi	0.022	0.023	0.011	0.016	0.016	0.016	0.023	0.023	0.027	0.029	0.041
Jiangsu	0.067	0.059	0.034	0.079	0.095	0.087	0.08	0.079	0.071	0.066	0.053
Anhui	0.064	0.067	0.048	0.041	0.041	0.038	0.043	0.05	0.058	0.057	0.048
Shanghai	0.014	0.031	0.029	0.027	0.048	0.049	0.057	0.044	0.044	0.043	0.034
Hubei	0.054	0.056	0.067	0.044	0.032	0.047	0.047	0.039	0.038	0.038	0.032
Sichuan	0.01	0.009	0.026	0.033	0.029	0.034	0.037	0.039	0.034	0.046	0.042
Zhejiang	0.062	0.051	0.048	0.049	0.054	0.035	0.029	0.035	0.04	0.037	0.03
Chongqing	0.04	0.022	0.039	0.019	0.015	0.014	0.017	0.017	0.014	0.013	0.011
Jiangxi	0.039	0.037	0.037	0.026	0.022	0.021	0.033	0.041	0.038	0.035	0.027
Hunan	0.063	0.085	0.079	0.067	0.05	0.044	0.038	0.043	0.055	0.055	0.048
Guizhou	0.022	0.024	0.022	0.031	0.023	0.032	0.028	0.032	0.032	0.03	0.024
Fujian	0.069	0.075	0.073	0.048	0.032	0.022	0.027	0.03	0.035	0.033	0.026

Variable	Influenza season										
	2005–06	2006–07	2007–08	2008–09	2009–10	2010–11	2011–12	2012–13	2013–14	2014–15	2015–16
Yunnan	0.045	0.054	0.041	0.028	0.028	0.051	0.042	0.03	0.037	0.049	0.039
Guangdong	0.09	0.061	0.091	0.084	0.082	0.088	0.074	0.072	0.071	0.066	0.052
Guangxi	0.07	0.054	0.045	0.026	0.034	0.032	0.025	0.033	0.044	0.04	0.033
Hainan	0.014	0.018	0.014	0.02	0.015	0.014	0.011	0.011	0.014	0.014	0.011
PCR result, %											
H1	0.321	0	0.6	0.021	0.002	0.001	0	0	0	0	0
H1N1pdm	0	0	0	0.056	0.159	0.051	0.002	0.038	0.048	0.001	0.049
H3	0	0.833	0	0.122	0.026	0.031	0.075	0.029	0.064	0.088	0.032
A(others)	0	0	0	0.063	0.02	0.006	0.006	0	0	0	0
B/Victoria	0	0	0	0.002	0.012	0.004	0.036	0.001	0.001	0.001	0.059
B/Yamagata	0	0.167	0.2	0.001	0.002	0.004	0.006	0.001	0.034	0.036	0.02
B(others)	0.226	0	0	0.012	0.055	0.025	0.067	0.002	0.013	0.005	0.016
Negative	0.452	0	0.2	0.724	0.724	0.878	0.807	0.928	0.838	0.867	0.824
Testing methods used by laboratories, %											
HAI assay	0.998	1	1	0.354	0.032	0.246	0.297	0.182	0.115	0.134	0.141
PCR	0.000	0	0	0.506	0.815	0.598	0.495	0.601	0.618	0.639	0.657
HAI assay and PCR	0.002	0	0	0.140	0.152	0.156	0.209	0.218	0.267	0.227	0.202

*Seasons defined as the start of October until the end of September of the next year. The column sum of proportions by age group, province/municipality, PCR result, and testing method = 1. The distribution of positive specimens identified by PCR by laboratories that adopted the PCR approach or both PCR and HAI assay for virus identification is shown under "PCR result," in which A(others) showed the proportion of specimens with unsubtyped or co-infected seasonal influenza A viruses and avian influenza viruses including H5-positive, H7-positive, H9-positive; whereas B(others) showed the proportion of specimen with unsubtyped or co-infected influenza B viruses. HAI, hemagglutination-inhibition.

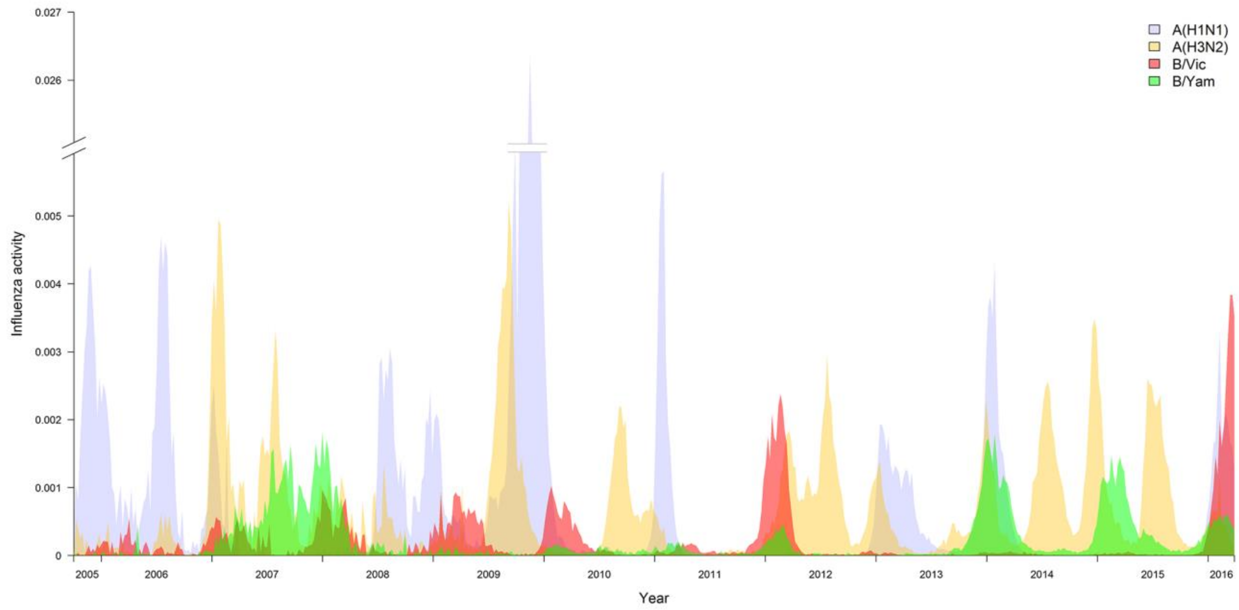

Technical Appendix Figure 1. National influenza virus activity by virus subtype (A(H1N1) and A(H3N2)) and lineage (B/Victoria and B/Yamagata), China, October 2005–March 2016. Findings are based on 2,498,735 specimens collected from the sentinel hospitals. Influenza activity is shown as the average of the provincial/municipal proxy, which is the product of the weekly proportion of influenza-like illness consultations and the weekly proportion of sentinel specimens testing positive for a specific type of virus, weighted by the population size of each province and municipality.

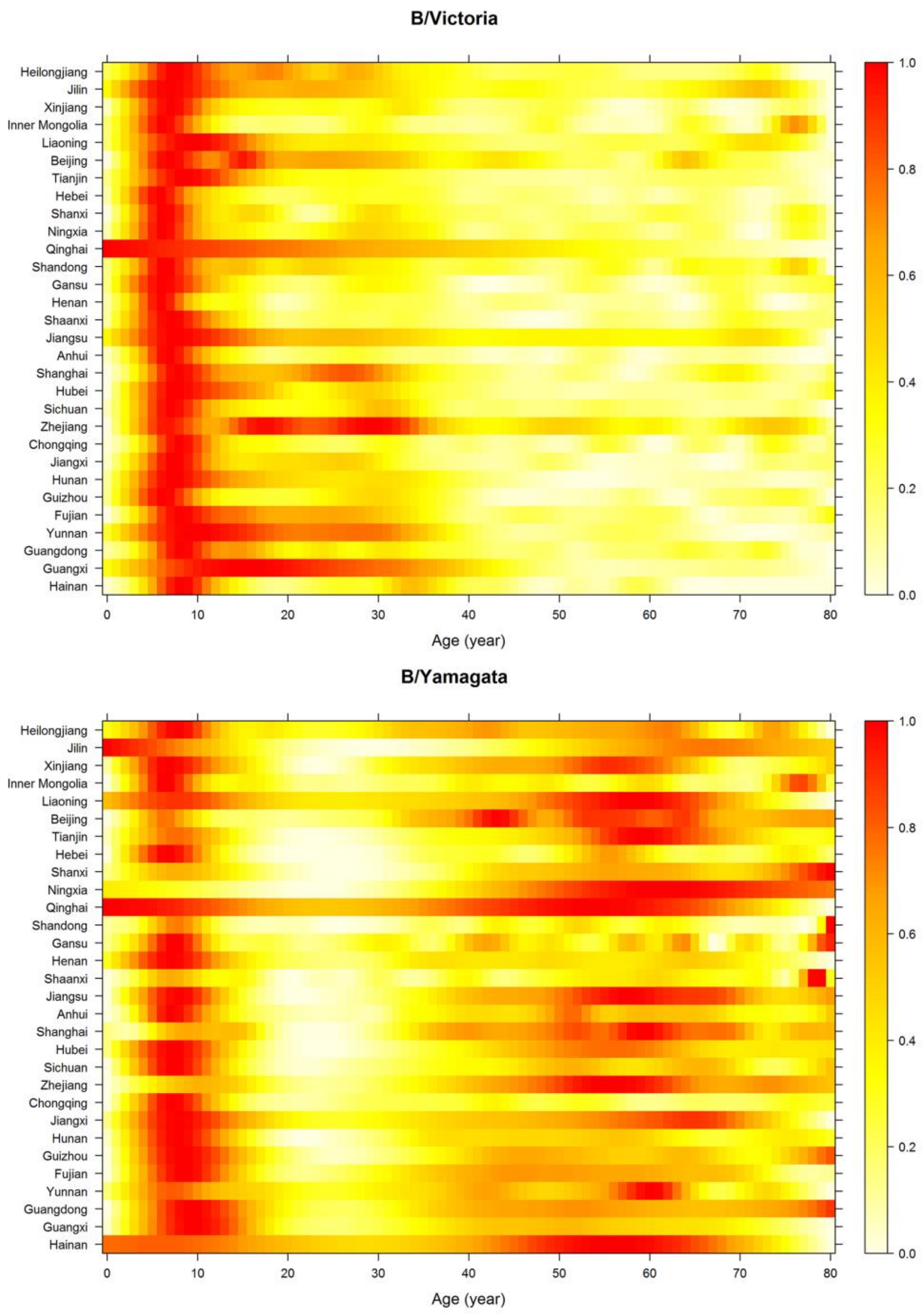

Technical Appendix Figure 2. Heatmap of the age-specific proportions of sentinel specimens testing positive for influenza B/Victoria and B/Yamagata lineages in 30 provinces and municipalities (sorted by latitude), China, October 2005–March 2016. Findings are based on 2,498,735 specimens collected from the sentinel hospitals. Normalized proportions are shown for each province and municipality as the smoothed age-specific proportions divided by the maximum smoothed proportion by lineage in the province and municipality throughout the study period to give a rescaled proxy with values between 0 (lowest proportion in the province over the study period) and 1 (highest proportion in that province over the study period).
